# Brain regulates weight bearing bone through PGE2 skeletal interoception: implication of ankle osteoarthritis and pain

**DOI:** 10.1038/s41413-024-00316-w

**Published:** 2024-03-05

**Authors:** Feng Gao, Qimiao Hu, Wenwei Chen, Jilong Li, Cheng Qi, Yiwen Yan, Cheng Qian, Mei Wan, James Ficke, Junying Zheng, Xu Cao

**Affiliations:** 1 Department of Orthopedic Surgery, Johns Hopkins University School of Medicine, Baltimore, Maryland 21205 USA; 2 Department of Biomedical Engineering, Johns Hopkins University School of Medicine, Baltimore, Maryland 21205 USA

**Keywords:** Bone, Pathogenesis

## Abstract

Bone is a mechanosensitive tissue and undergoes constant remodeling to adapt to the mechanical loading environment. However, it is unclear whether the signals of bone cells in response to mechanical stress are processed and interpreted in the brain. In this study, we found that the hypothalamus of the brain regulates bone remodeling and structure by perceiving bone prostaglandin E2 (PGE2) concentration in response to mechanical loading. Bone PGE2 levels are in proportion to their weight bearing. When weight bearing changes in the tail-suspension mice, the PGE2 concentrations in bones change in line with their weight bearing changes. Deletion of *cyclooxygenase-2 (COX2)* in the osteoblast lineage cells or knockout of receptor 4 (*EP4)* in sensory nerve blunts bone formation in response to mechanical loading. Moreover, knockout of *TrkA* in sensory nerve also significantly reduces mechanical load-induced bone formation. Moreover, mechanical loading induces cAMP-response element binding protein (CREB) phosphorylation in the hypothalamic arcuate nucleus (ARC) to inhibit sympathetic tyrosine hydroxylase (TH) expression in the paraventricular nucleus (PVN) for osteogenesis. Finally, we show that elevated PGE2 is associated with ankle osteoarthritis (AOA) and pain. Together, our data demonstrate that in response to mechanical loading, skeletal interoception occurs in the form of hypothalamic processing of PGE2-driven peripheral signaling to maintain physiologic bone homeostasis, while chronically elevated PGE2 can be sensed as pain during AOA and implication of potential treatment.

## Introduction

Life started on Earth about 3.7 billion years ago, only several hundred million years after the birth of Earth^1^. It took 3 billion years of molecular and cellular evolution to develop nerve circuits of the brain. The emerge of the brain brought quick evolution of various species in the animal kingdom from ocean to land including aquatic vertebrates, amphibians, birds, and mammals in the last 600 million years^2–5^. Interoception is a type of brain circuits that monitor the organism’s internal state to regulate the complex interactions between the brain and peripheral organs^6,7^. The signals collected from sensory nerve endings of peripheral tissues, such as skin, joint, respiratory, and gastrointestinal tissues, are processed in the central nervous system (CNS) to initiate physiological responses. The hypothalamus controls whole-body homeostasis by integrating peripheral information and coordinating peripheral organs through descending neural or neuroendocrine pathways^8^. We have recently established that skeletal interoception regulates bone homeostasis. The sensory nerve is activated by prostaglandin E2 (PGE2) in bone to induce phosphorylation of cAMP-response element binding protein (CREB) in the hypothalamus as ascending interoceptive signal, which tunes down sympathetic activity for osteoblastic bone formation as the descending interoceptive signal^9,10^. Importantly, the skeletal interoceptive signal also downregulates hypothalamic Neuropeptide Y (NPY) expression to induce adipose tissue lipolysis for osteoblastic bone formation^11^. Moreover, skeletal interoception promotes biomaterial-mediated new bone formation through divalent metal cations stimulation of macrophage secretion of PGE2 pain^12^. These results suggest that PGE2 skeletal interoception regulates bone quality in response to mechanical loading.

The primary functions of the skeleton of terrestrial animals are to provide mechanical support for locomotion and physical activity, to protect fragile organs and to serve as a metabolic mineral reserve^13^. The mechanical properties of bone are dependent on its microarchitecture and its integrity is critical to prevent fracture^14^. PGE2 is considered a potent anabolic regulator of bone growth. The PGE2 level increases in loaded bone tissue, and direct administration results in bone formation^15^. PGE2 administration also increases the sensitivity of bone to mechanical loading^16^. Biochemical blockage of prostaglandins leads to bone’s inability to sense and respond to mechanical stimulation^17^. Moreover, osteoblasts subjected to fluid shear increase the expression of cyclooxygenase-2 (COX2), the enzyme responsible for PGE2 synthesis^18^. Therefore, PGE2 could serve as a mechanotransduction signal that translates the external mechanical load signal into biochemical responses through skeletal interoception.

Bone as a mechanosensitive tissue undergoes constant remodeling to adapt to the physical environment, including loading-induced microfractures that repeatedly occur in response to gravity and subsequent weight bearing^19^. Mechanical loads increase bone formation by stimulating the activity of bone-forming osteoblasts^20^. In contrast, loss of mechanical loads induces negatively balanced bone remodeling, resulting in more bone resorption than bone formation, like astronauts in space^21^. Bone cells are tightly coupled to their extracellular environment and coordinate the bone formation process by converting external mechanical load into biochemical responses as mechanotransduction^22^. The external mechanical load generates deformation in the bone tissue, which results in changes in whole tissue strain, shear stress, hydrostatic pressure, and streaming potentials generated by bone fluid flow^23^. Molecular and cellular mechanisms that mediate mechanotransduction have been studied extensively in various experimental models^24–27^.In addition, the endocrine regulation of bone metabolism, including the balance between osteoclast-mediated bone catabolism and osteoblast-induced bone anabolism, has also been extensively studied in recent decades^28–31^. However, it is still unclear whether the signals derived from bone cells in response to mechanical stress are processed and interpreted in the brain to regulate bone remodeling to maintain its mechanical structure and metabolism and how this might be altered during disease. Aberrant mechanical loading induces uncoupled bone remodeling to generate porosity structure of the spine endplate for low back pain and porous subchondral bone for osteoarthritis pain^32–34^. The talus in the ankle bears the highest body weight^35^. Ankle osteoarthritis (AOA) is known to be very painful^36^. Currently, there is no disease-modifying treatment for the joint pain orders, leaving surgical treatments such as joint replacement as the only approach for the end stage of these diseases^37–39^.

In this study, we investigated whether the PGE2 skeletal interoception in response to mechanical stress is processed during normal physiology and how the brain interprets the signaling. We found that the density of different bones in the body is proportionally correlated to PGE2 concentrations in the mouse bone. Notably, we found that the talus, which bears more body weight than any other weight-bearing bones during movement or physical activities, has the highest PGE2 levels. Mechanistically, we found that mechanical loading increased osteogenesis by increasing CREB phosphorylation in the arcuate nucleus (ARC) of the hypothalamus, which resulted in the suppression of sympathetic tone. On the molecular level, such PGE2-driven interoception resulted in an elevation of the cAMP response element modulator (CREM), the endogenous repressor of cAMP-responsive element (CRE)-mediated gene transcription, to suppress TH gene expression and thus sympathetic tone. Finally, we found that elevated PGE2 levels are positively associated with AOA-related pain. COX2 expression in the talus was extremely high, which explains painful AOA and PGE2 in control of bone quality and skeletal pain.

## Results

### PGE2 concentrations in different bones are positively correlated with their weight bearing

**Fig. 1:**
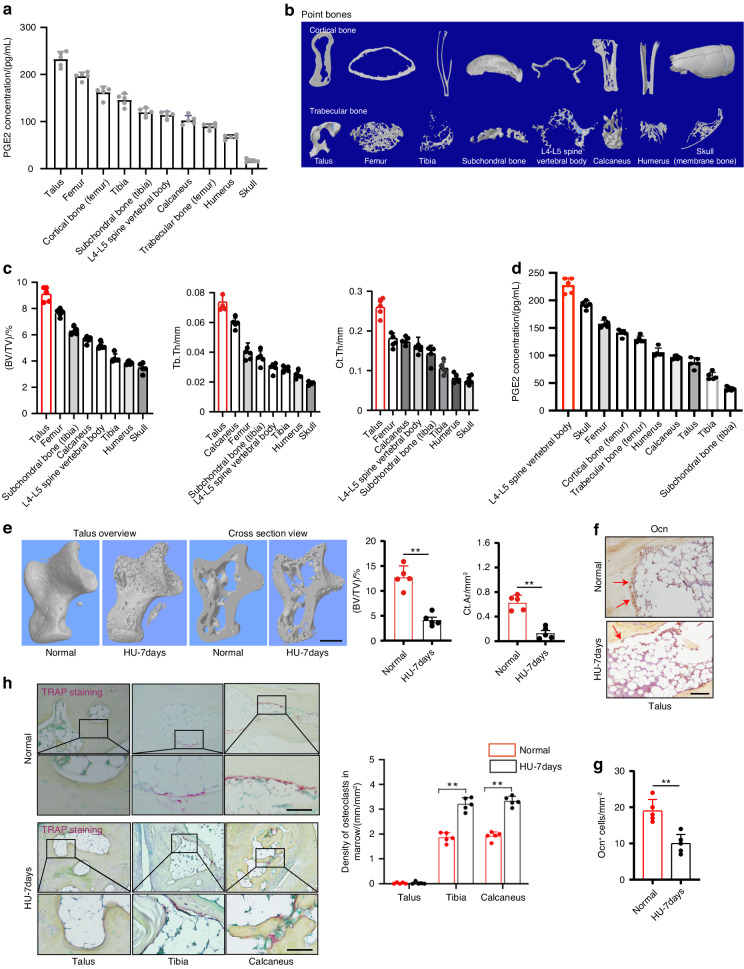
PGE2 concentration in bone is positively correlated with mechanical load. **a** Enzyme-linked immunosorbent assay (ELISA) analysis of PGE2 level in bone at 10 different points of 12-week-old *C57BL/6* mice. **b** Representative μCT images of 8 different point bones from 12-week-old *C57BL/6* mice. **c** Quantitative analysis of trabecular bone fraction (BV/TV), trabecular bone thickness (Tb. Th) and cortical bone thickness (Ct. Th). **d** ELISA analysis of PGE2 level in bone at 10 different points of 13-week-old *C57BL/6* mice with HU for 7 days. **e** Representative μCT images and quantitative analysis of trabecular bone fraction (BV/TV), cortical bone area (Ct. Ar) of talus from normal 13-week-old *C57BL/6* mice or with HU for 7 days. Scale bars, 50 μm. Representative images of immunostaining of osteocalcin (Ocn) positive cells **f** and analysis of Ocn^+^ cells in the subchondral bone of talus from normal 13-week-old *C57BL/6* mice or with HU for 7 days **g**. Scale bars, 50 μm. **h** Representative images of immunostaining and quantitative analysis of the density of TRAP^+^ cells in the subchondral bone of talus, tibia, and calcaneus from normal 13-week-old *C57BL/6* mice or with HU for 7 days. Scale bars, 50 μm. *n* ≥ 5 per group. *P < 0.05, **P < 0.01, and N.S. indicates not significant. Statistical significance was determined by Student’s *t*-test

Mechanical loading stimulates an increase in PGE2 levels in the skeletal system. To investigate the role of this increase in mechanical loading, we measured PGE2 levels in the bone at ten different locations of *C57BL/6* mice at 12 weeks of age. Interestingly, PGE2 levels in the talus were the highest and lowest in the skull (Fig. 1a). Analysis by in vitro microcomputerized tomography (μCT) of bones at different locations within the body harvested at 12 weeks of age revealed that the talus had the highest bone volume fraction (BV/TV), as well as the trabecular thickness (Tb.Th) and cortical thickness (Ct.Th), which are indices of trabecular and cortical structure, respectively, while the skull had the lowest of these three parameters. (Fig. 1b, c). Together, these findings suggest that the concentration of PGE2 in each site of bone is proportional to its degree of weight bearing.

To determine whether the change of mechanical loading in the skeleton affects PGE2 secretion to regulate bone homeostasis, hindlimb unloading (HU) was investigated. HU is a ground‐based model that creates hypokinesia and hypodynamia at the hind limbs and mimics cephalic fluid‐shift aspects of space flight. Extensive studies in rats showed that it induces both cortical and cancellous bone loss in the hind limbs^40^. We thus applied HU to *C57B/L6* mice and measured bone PGE2 levels. We found that PGE2 levels significantly decreased in the talus compared with the grounded control group, while it increased in the skull and spine (Fig. 1d). Bone mass and cortical bone of the talus decreased significantly in HU mice while body weight remained unchanged over time relative to their grounded littermate controls (Fig. 1e)^41^. Again, the number of osteoblasts in the talus decreased significantly in the HU mice compared to the controls (Fig. 1f, g), whereas the number of tartaric acidic phosphatase (TRAP^+^) osteoclasts in the talus was not detected either in the ground control or the HU mice (Fig. 1h). As PGE2 is predominantly secreted by osteoblasts, the decrease of osteoblasts is consistent with the decrease of PGE2 levels in the talus after 7 days of HU.

### PGE2 concentrations in the bone increase in response to mechanical loading to regulate bone formation

**Fig. 2:**
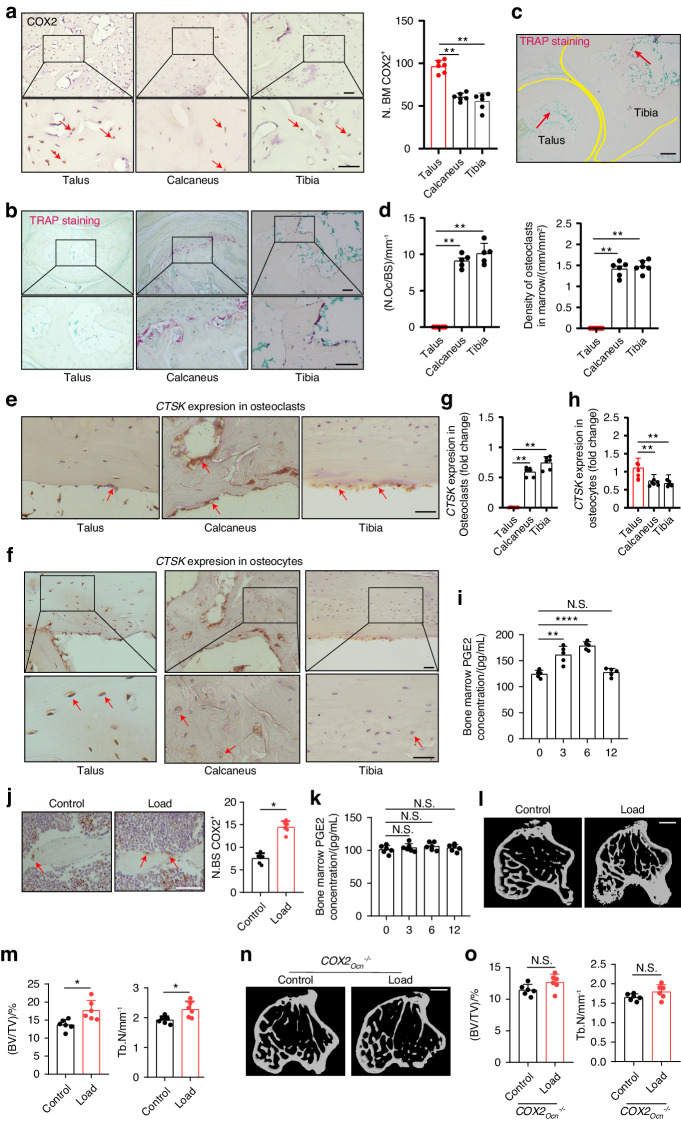
PGE2 mediates mechanical load-induced osteoblastic bone formation. **a** Representative images of immunostaining and quantitative analysis of the number of COX2^+^ cells (brown) in the subchondral bone marrow (N.BM/COX2^+^) of talus, calcaneus, and tibia from 12-week-old C57BL/6 mice. Scale bars, 50 μm. Representative images of immunostaining TRAP^+^ cells per bone surface (N.Oc/BS) on the trabecular bone surface **b** and TRAP^+^ cells in the bone marrow **c** and quantitative analysis of TRAP^+^ cells **d** in talus, calcaneus, and tibia from 12-week-old *C57BL/6* mice. Scale bars, 50 μm. Representative images of immunostaining **e** and quantitative analysis **g** of CTSK expression in osteoclasts at steady state of the subchondral bone of talus, calcaneus, and tibia from 12-week-old *C57BL/6* mice. Scale bars, 50 μm. Representative images of immunostaining **f** and quantitative analysis **h** of CTSK expression in osteocytes at steady state of the subchondral bone of talus, calcaneus, and tibia from 12-week-old *C57BL/6* mice. Scale bars, 50 μm. **i** ELISA analysis of PGE2 level in tibiae bone marrow at different time points after axial compression loading on the tibiae of WT mice. **j** Immunohistochemical staining and quantification of COX2^+^ cells (brown) on the trabecular tibial surface in WT mice. Scale bar, 50 µm. **k** ELISA analysis of PGE2 level in tibiae bone marrow at different time points after axial compression loading on the tibiae 100 cycles at 2 Hz of *COX2*_*Ocn*_^*−/−*^ mice. Representative µCT images **l** and quantitative analysis **m** of trabecular bone fraction (BV/TV) and trabecular number (Tb.N) of tibial bone of WT mice loaded for one month or non‐loaded tibiae. Scale bar, 500 µm. Representative µCT images **n** and quantitative analysis **o** of trabecular bone fraction (BV/TV) and trabecular number (Tb.N) of tibial bone of *COX2*_*Ocn*_^*−/−*^ mice loaded for one month or non‐loaded tibiae. Scale bar, 500 µm. *n* ≥ 5 per group. *P < 0.05, **P < 0.01, and N.S. indicates not significant. Statistical significance was determined by Student’s *t*-test

We next sought to examine whether COX2, which is the key rate-limiting biosynthetic enzyme for PGE2, mediates bone formation. By analysis of COX2 expression levels, we found significantly higher expression in the talus than in the tibia and calcaneus (Fig. 2a). Of note, we found that there was practically no TRAP staining in the talus while there was relatively strong staining in the tibia and calcaneus of adult mice (Fig. 2b), which correlated with a strong difference in the number of osteoclasts per bone surface (N.Oc/BS) and the density of osteoclasts in the bone marrow of these bones (Fig. 2c, d). We also found that cathepsin K (CTSK), which is highly expressed and secreted by osteoclasts and is required for their bone-resorbing activity, is also expressed in osteocytes (Fig. 2f). Notably, we observed a considerable number of CTSK^+^ osteoclasts at steady state in the calcaneus and tibia, but not in the talus (Fig. 2e, g), while immunohistochemistry revealed CTSK^+^ osteocytes at steady state in the talus, calcaneus and tibia (Fig. 2f, h). These results likely indicate that CTSK in osteocytes regulates talus bone surface remodeling. These findings provide evidence that under physiological conditions, PGE2 in the talus mediates bone remodeling independent of osteoclasts.

PGE2 is known to stimulate osteoblastic bone formation. We measured PGE2 levels in the tibia bone marrow (Fig. 2i) following axial tibial compression. We found that PGE2 levels increased at 3 and 6 h after loading and then dropped to normal levels after 12 h (Fig. 2i). By immunohistochemical analysis we also found that expression of COX2 was higher on the trabecular surface of the tibial bone after loading compared to the non-loading control (Fig. 2j). In addition, by μCT analysis we found that there was a significantly greater degree of bone formation after loading for 1 month (Fig. 2l, m).

To further examine whether PGE2 is secreted primarily by osteoblastic cells in response to mechanical loading, we generated conditional knockout *COX2* mice in osteoblastic cells (*COX2*_*Ocn*_^*−/−*^) by crossing *COX2* floxed (*COX2*^*wt*^) mice with osteocalcin (*Ocn*)-Cre mice to eliminate PGE2 secretion by osteoblastic cells as Ocn is a selective marker of osteoblasts. Bone PGE2 concentrations were not changed after loading within 12 h in the *COX2*_*Ocn*_^*−/−*^ mice, suggesting that PGE2 was largely secreted by osteoblasts (Fig. 2k). Moreover, we did not see an increase in bone formation in *COX2*_*Ocn*_^*−/−*^ mice after mechanical loading (Fig. 2n, o) suggesting that mechanical loading via upregulation of PGE2 levels promotes bone formation.

### Deletion of *TrkA* in sensory nerve reduces talus bone formation

**Fig. 3:**
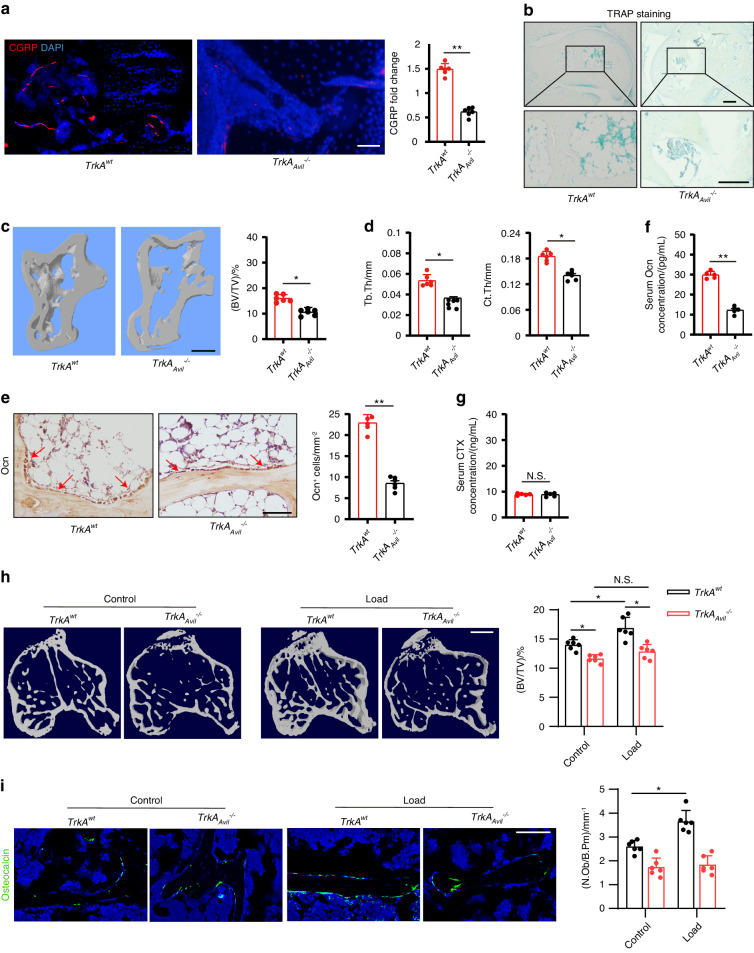
Deletion of *TrkA* in sensory nerve reduces mechanical load-induced bone formation. **a** Representative images of immunofluorescence staining and quantitative analysis of the CGRP^+^ sensory nerves (red) in the subchondral bone of talus from 12-week-old *TrkA*^*wt*^ and *TrkA*_*Avil*_^*−/*−^ mice. Scale bar, 100 μm. **b** Representative images of immunostaining of TRAP^+^ cells in the subchondral bone of talus from 12-week-old *TrkA*^*wt*^ and *TrkA*_*Avil*_^*−/−*^ mice. Scale bar, 100 μm. Representative μCT images and quantitative analysis of trabecular bone fraction (BV/TV) **c**, trabecular bone thickness (Tb. Th) and cortical bone thickness (Ct. Th) **d** of talus from 12-week-old *TrkA*^*w*t^ and *TrkA*_*Avil*_^*−/−*^ mice. Scale bar, 100 μm. **e** Representative images of immunostaining of Ocn and analysis of Ocn^+^ cells in the subchondral bone of talus from 12-week-old *TrkA*^*wt*^ and *TrkA*_*Avil*_^*−/−*^ mice. Scale bar, 100 μm. ELISA analysis of Ocn **f** and carboxy-terminal collagen crosslinks (CTX) level of the serum **g** from 12-week-old *TrkA*^*w*t^ and *TrkA*_*Avil*_^*−/−*^ mice. *TrkA*^*wt*^ and *TrkA*_*Avil*_^*−/−*^ mice underwent one month of axial compression loading of tibiae. Non‐loaded tibiae were used as controls. **h** Representative µCT images and quantitative analysis of trabecular bone fraction (BV/TV) of tibial bone. Scale bar, 500 µm. **i** Representative images of immunofluorescence staining and quantitative analysis of Ocn^+^ cells (green) on trabecular bone surface of tibiae. Scale bar, 50 μm. *n* ≥ 5 per group. *P < 0.05, **P < 0.01, and N.S. indicates not significant. Statistical significance was determined by Student’s *t*-test

To investigate the role of sensory nerves in talus homeostasis, we crossed nerve growth factor receptor *TrkA* floxed (*TrkA*^*wt*^) mice with sensory neuron-specific Cre mice (*Advilin-Cre*) to generate mice with deletion of *TrkA* in sensory nerve (*TrkA*_*Avil*_^*–/–*^). Immunostaining of talus sections showed that most calcitonin gene-related peptide (CGRP^+^) sensory nerve fibers were eliminated in the *TrkA*_*Avil*_^*–/–*^mice (Fig. 3a). TRAP staining showed no osteoclast formation in either the talus of the *TrkA*^*wt*^ or *TrkA*_*Avil*_^*–/–*^ mice (Fig. 3b). By μCT analysis we found that there was significantly less bone in talus in 12-week-old *TrkA*_*Avil*_^*–/–*^ mice relative to their wild-type (WT) littermates (Fig. 3c). In addition, Tb.Th and Ct.Th were lower in *TrkA*_*Avil*_^*–/–*^ mice compared to *TrkA*^*wt*^ mice indicating an essential role of sensory nerve function for talus homeostasis in adults (Fig. 3d). Notably, the number of Ocn^+^ osteoblasts and the serum levels of Ocn were significantly lower in *TrkA*_*Avil*_^*–/–*^ mice relative to their wild-type littermates (Fig. 3e, f), while the level of the osteoclast bone resorption marker, carboxy-terminal collagen crosslinks (CTX), was not different in *TrkA*_*Avil*_^*–/–*^ mice compared to *TrkA*^*wt*^ control (Fig. 3g).

### Deletion of *TrkA* in sensory nerve reduces mechanical loading-induced bone formation

We next tested whether deletion of *TrkA* in sensory nerve could reduce mechanical loading-induced bone formation. *TrkA*^*wt*^ and *TrkA*_*Avil*_^*−/−*^ mice underwent one month of axial compression loading of tibiae. In the *TrkA*^*wt*^ mice, mechanical load significantly increased the bone formation in the tibiae compared to the non‐loading controls (Fig. 3h). The load-induced bone formation was abolished in the *TrkA*_*Avil*_^*–/–*^ mice (Fig. 3h). In addition, immunostaining of trabecular bone sections showed that Ocn^+^ osteoblastic cells were significantly greater in wild-type mice after mechanical loading compared to the non-loading control, and no such increase occurred in *TrkA*_*Avil*_^*–/–*^ mice (Fig. 3i). Taken together, these results indicate that peripheral sensory nerves regulate load-induced osteogenesis.

### Mechanical loading induces osteogenesis via central CREB signaling

**Fig. 4:**
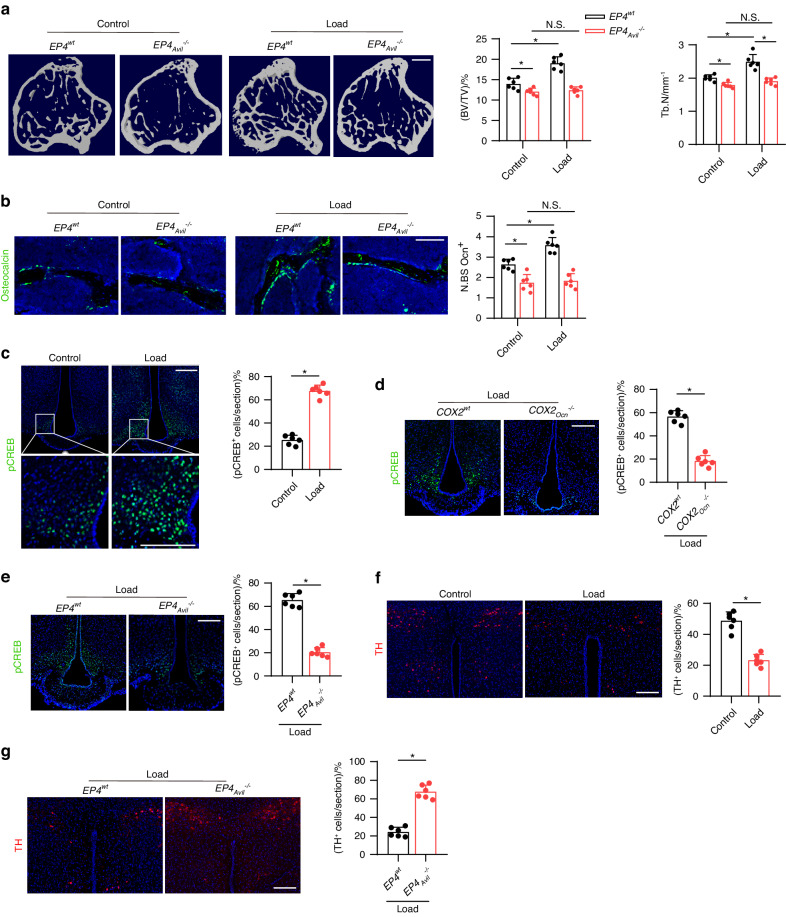
Mechanical load induces osteogenesis through PGE2/EP4 interoceptive signaling. *EP4*^*wt*^ and *EP4*_*Avil*_^*−/−*^ mice underwent one month of axial compression loading of tibiae. Non‐loaded tibiae were used as controls. **a** Representative µCT images and quantitative analysis of trabecular bone fraction (BV/TV) and trabecular number (Tb.N) of tibial bone. Scale bar, 500 µm. **b** Representative images of immunofluorescence staining of Ocn and quantitative analysis of Ocn^+^ cells (green) on trabecular bone surface of tibiae. Scale bar, 50 μm. **c** Representative images of immunofluorescence staining and quantitative analysis of the pCREB^+^ cells in the ARC of the hypothalamus of WT mice underwent three consecutive days of axial compression loading of tibiae or control sham load. Scale bar, 50 μm. **d** Representative images of immunofluorescence staining and quantitative analysis of the pCREB^+^ cells in the ARC of the hypothalamus of *COX2*^*wt*^ and *COX2*_*Ocn*_^*−/−*^ mice underwent three consecutive days of axial compression loading of tibiae. Scale bar, 50 μm. **e** Representative images of immunofluorescence staining and quantitative analysis of the pCREB^+^ cells in the ARC of the hypothalamus of *EP4*^*wt*^ and *EP4*_*Avil*_^*−/−*^ mice underwent three consecutive days of axial compression loading of tibiae. Scale bar, 50 μm. **f** Representative images of immunofluorescence staining and quantitative analysis of the TH^+^ cells in the PVN of the hypothalamus of WT mice underwent three consecutive days of axial compression loading of tibiae or control sham load. Scale bar, 50 μm. **g** Representative images of immunofluorescence staining and quantitative analysis of the TH^+^ cells in the PVN of the hypothalamus of *EP4*^*wt*^ and *EP4*_*Avil*_^*−/−*^ mice underwent three consecutive days of axial compression loading of tibiae. Scale bar, 50 μm. *n* ≥ 5 per group. *P < 0.05, **P < 0.01, and N.S. indicates not significant. Statistical significance was determined by Student’s *t*-test

The PGE2 receptor 4 (EP4) receptor is a key receptor for PGE2 in peripheral sensory nerves, and its signaling maintains the balance of osteogenesis and adipogenesis in adult mice by regulating sympathetic nerve activity^10^. To determine whether mechanical loading-induced bone formation is mediated by PGE2-EP4 signaling in sensory nerves, we generated sensory nerve-specific EP4 knockout mice (*EP4*_*Avil*_^–/–^) by crossing *EP4 floxed (EP4*^*wt*^) mice with *Advilin-Cre* mice. In the *EP4*^*wt*^ mice, the bone mass increased significantly after mechanical loading compared to the control group, as shown by μCT analysis. However, this effect was diminished in the *EP4*_*Avil*_^–/–^ mice (Fig. 4a). Similarly, by immunostaining of trabecular bone sections we found that mechanical loading was associated with a significantly greater number of Ocn^+^ osteoblastic cells in *EP4*^*wt*^ mice compared to the non-loading control and these effects were absent in *EP4*_*Avil*_^–/–^ mice (Fig. 4b).

Osteoblast-derived PGE2-mediated activation of EP4 receptors in peripheral sensory nerves promotes bone formation by inhibiting sympathetic activity via hypothalamic CREB signaling^9^. By immunostaining of hypothalamic sections, we found that mechanical loading was associated with significantly greater levels of phosphorylated CREB (pCREB) in the ARC compared with the non-loading control group (Fig. 4c). In contrast, the hypothalamic pCREB levels in the ARC in the *COX2*_*Ocn*_^*−/−*^ mice were not greater after mechanical loading compared with the *COX2*^*wt*^ mice (Fig. 4d). These data further demonstrate that osteoblasts secrete PGE2 in response to mechanical loading to activate skeletal interoception. Moreover, in the *EP4*_*Avil*_^–/–^ mice, the hypothalamic pCREB levels in the ARC were not higher after mechanical loading compared with *EP4*^*wt*^ control mice (Fig. 4e), suggesting that mechanical loading may stimulate bone formation by activating a PGE2-EP4 ascending interoceptive pathway.

To evaluate sympathetic nerve activity, we performed immunostaining for tyrosine hydroxylase (TH), the rate-limiting enzyme in the synthesis of catecholamines, in the paraventricular nucleus (PVN) of the hypothalamus. TH expression was significantly lower in the mechanical loading group compared to the control group (Fig. 4f). As expected, mechanical loading was associated with lower TH expression in the PVN of the *EP4*^*wt*^ mice but not the *EP4*_*Avil*_^–/–^mice (Fig. 4g). Taken together, these findings suggest that skeletal interoception in response to mechanical loading is induced by CREB phosphorylation in the ARC, which inhibits TH expression in the PVN to promote osteogenesis.

### Mechanical loading signals through the ARC to regulate sympathetic activity

**Fig. 5:**
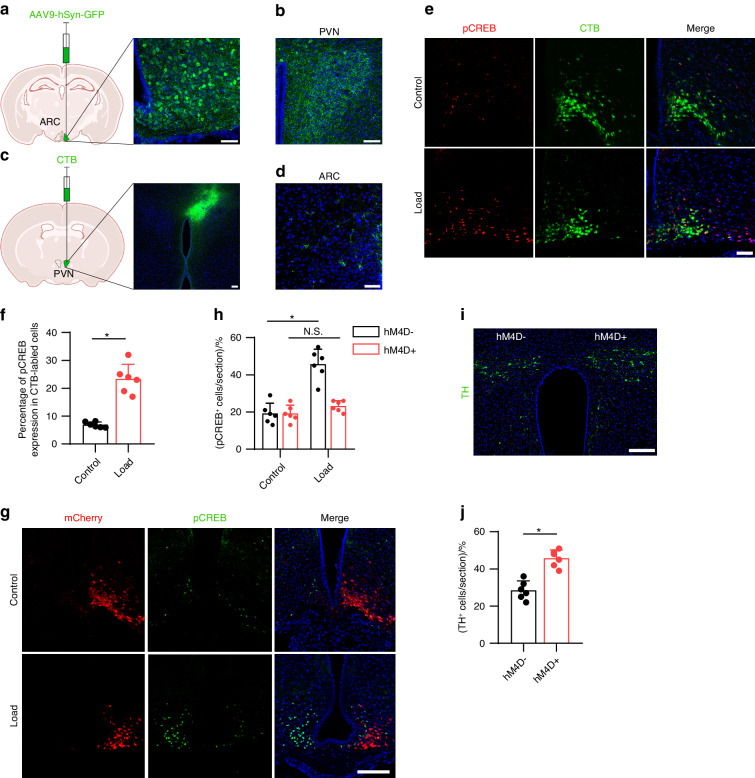
Mechanical loading regulates sympathetic activity through hypothalamus AgRP neurons. **a** Diagram of the AAV9-hSyn-GFP injection sites in the ARC area of the WT mice. **b** Representative images of GFP^+^ neurons in the PVN of the hypothalamus AAV9-hSyn-GFP injection in ARC. **c** Diagram of the CTB injection site in the PVN area of the WT mice. **d** Representative images of GTB^+^ neurons in the ARC of the hypothalamus after CTB injection in PVN. Representative images of immunofluorescence staining **e** and quantitative analysis of the pCREB (red) and CTB (green) **f** in the ARC of the hypothalamus of WT mice underwent three consecutive days of axial compression loading of tibiae or control sham load after CTB injection in the PVN for 5 days. Scale bar, 50 μm. *AgRP-Ires-Cre* mice injected with pAAV-hSyn-DIO-hM4d(Gi)-mCherry in one side of the ARC (right) and control AAV in the other side (left). Mice injected saline or CNO (0.3 mg/kg of body weight, i.p.) before loading. Representative images of immunofluorescence staining **g** and quantitative analysis **h** of the pCREB (green) and mCherry (red) in the ARC of hypothalamus of *AgRP-Ires-Cre* mice underwent three consecutive days of axial compression loading of tibiae or control sham load. Scale bar, 50 μm. Representative images of immunofluorescence staining **i** and quantitative analysis of the TH (green) **j** in the PVN of hypothalamus of *AgRP-Ires-Cre* mice that underwent three consecutive days of axial compression loading of tibiae. Scale bar, 50 μm. *n* ≥ 5 per group. *P < 0.05, **P < 0.01, and N.S. indicates not significant. Statistical significance was determined by Student’s *t*-test

The ARC is an essential site for the control of energy balance and the autonomic nervous system. The PVN has been identified as the primary hypothalamic site that contributes to the control of sympathetic outflow and energy expenditure^42^. In the ARC, we have previously found that stimulation of skeletal interoception downregulates hypothalamic NPY expression to mediate lipolysis in the adipose tissue to liberate fatty acids to provide fuel for anabolic bone formation. To demonstrate that the ARC-PVN neural circuit controls energy balance, we first injected anterograde viral tracer AAV9-hSyn-GFP into the ARC of mice brain and found that neural fibers in PVN were labeled by GFP (Fig. 5a, b). Meanwhile, we injected the retrograde neural tracer cholera toxin B subunit (CTB) into the PVN of mice and found many CTB-labeled neurons in the ARC (Fig. 5c, d). These data suggested that ARC neurons project to the PVN. More importantly, mechanical loading was associated with significantly greater expression of pCREB in CTB-labeled neurons in the ARC compared to the non-loading control group (Fig. 5e, f). This combined data suggests an ARC-PVN circuit is involved in mechanical loading-induced skeletal interoception.

Agouti-related peptide (AgRP^+^) neurons, which are primarily located in the ARC, express NPY and gamma-aminobutyric acid (GABA) to decrease energy expenditure via the sympathetic nervous system (SNS)^43^. To test whether AgRP^+^ neuron activity is sufficient to inhibit TH expression in the PVN, we chemogenetically inhibited AgRP^+^ neurons during mechanical loading by injecting a Cre-dependent adeno-associated virus (pAAV-hSyn-DIO-hM4d(Gi)-mCherry) unilaterally into the ARC. The infected AgRP^+^ neurons express inhibitory designer receptors hM4d, which can be inhibited following injection of the designer ligand clozapine-*N*-oxide (CNO). Inhibition of AgRP^+^ neurons on the right side of the brain was associated with significantly lower pCREB expression in the ARC during mechanical loading compared with the control left side of the brain (Fig. 5g, h). Importantly, the suppression of TH expression in the PVN after mechanical loading was abolished in the AAV injection side (right side) in the ARC compared with the control (left) side (Fig. 5i, j). Thus, these results suggest that mechanical loading activates an ARC-PVN circuit to tone down sympathetic nerve activity.

### Mechanical loading induces expression of the transcriptional repressor CREM via a PGE2-EP4 ascending interoceptive pathway

**Fig. 6:**
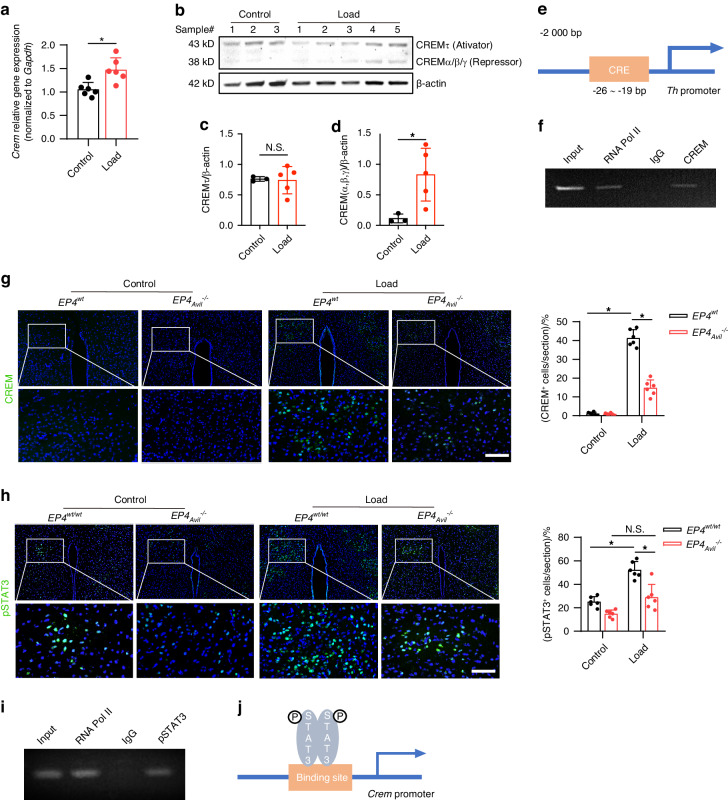
Mechanical load suppresses sympathetic activity through the ARC → PVN circuit via PGE2/EP4 interoceptive pathway. **a** RT-PCR quantitative analysis of *Crem* gene expression in the PVN area of the hypothalamus of WT mice underwent three consecutive days of axial compression loading of the tibia or control sham load. **b** Western blot analysis of CREMτ and CREMα/β/γ isoforms in the PVN area of the hypothalamus of WT mice underwent three consecutive days of axial compression loading of tibiae (*n* = 5) or control sham load (*n* = 3). **c**, **d** Quantitative analysis of CREMτ and CREMα/β/γ from Western blot in **b**. **e** Diagram of potential CREM binding site on the TH gene promoter. **f** ChIP analysis of CREM on TH gene promoter in the PVN area of WT mice underwent three consecutive days of axial compression loading of the tibia. **g** Representative images of immunofluorescence staining and quantitative analysis of the CREM^+^ cells in the PVN of the hypothalamus of *EP4*^*wt*^ and *EP4*_*Avil*_^*−/−*^ mice underwent three consecutive days of axial compression loading of tibiae or control sham load. Scale bar, 50 μm. **h** Representative images of immunofluorescence staining and quantitative analysis of the pSTAT3^+^ cells in the PVN of the hypothalamus of *EP4*^*wt*^ and *EP4*_*Avil*_^*−/−*^ mice underwent three consecutive days of axial compression loading of tibiae or control sham load. Scale bar, 50 μm. *n* ≥ 5 per group. *P < 0.05, **P < 0.01, and N.S. indicates not significant. Statistical significance was determined by Student’s *t*-test for A. Statistical significance was determined by two-way analysis of variance for **e**, **f**. **i** ChIP analysis of pSTAT3 on CREM gene promoter in the PVN area of WT mice underwent three consecutive days of axial compression loading of tibiae. **j** Diagram of the mechanism of mechanical load up-regulated CREM gene expression in the PVN area

The CREM, encoded by the *Crem* gene, is an endogenous modulator of CRE-mediated gene transcription. The *Crem* gene is notable for its production of multiple alternatively spliced transcript variants, including CREMτ, CREMα, CREMβ, CREMγ, and ICER, each with distinct functionalities. CREMτ functions as a transcriptional activator, whereas CREMα, CREMβ, CREMγ, and ICER typically act as transcriptional repressors^44^. To determine whether mechanical loading increases the expression of CREM in the hypothalamus to regulate *Th* gene expression, we measured *Crem* mRNA expression in the PVN in mice after mechanical loading through reverse transcription-polymerase chain reaction (RT-PCR). *Crem* mRNA expression was significantly higher in loaded mice compared to the non-loading control group (Fig. 6a). By Western blot analysis, we also found greater expression level of transcriptional suppressive CREMα, β, and γ, rather than CREMτ, in the hypothalamus of loaded mice than in the non-loading control mice (Fig. 6b–d). To investigate the transcriptional mechanism regulating this difference, we performed chromatin immunoprecipitation (ChIP) assay on the CRE binding site in the *Th* gene promoter (Fig. 6e) and found that mechanical loading induced specific binding of CREM to the CRE binding site of the *Th* promoter (Fig. 6f). Moreover, to examine whether mechanical loading induced CREM expression through the PGE2-EP4-mediated skeletal interoception pathway, we analyzed the levels of CREM expression in the PVN of loaded and control in the *EP4*_*Avil*_^–/–^ and *EP4*^*wt*^ mice. By immunostaining of hypothalamic sections, we found that the expression of CREM was significantly greater in the PVN of loaded *EP4*^*wt*^ mice compared to the non-loading *EP4*^*wt*^ group. And the load-induced increase of CREM expression was drastically reduced in the *EP4*_*Avil*_^–/–^ mice (Fig. 6g). Taken together, these results indicate that mechanical loading activates PGE2-EP4-mediated skeleton interoceptive signaling via an ARC-PVN circuit to induce CREM expression in the PVN to suppress TH expression and, thus, sympathetic drive.

Phosphorylated STAT3 (p-STAT3) acts as a transcription factor that binds to and regulate the expression of target genes, such as *proopiomelanocortin (POMC)*, to regulate energy metabolism. We found that mechanical loading induced significant greater levels of p-STAT3 in the PVN in the *EP4*^*wt*^ mice (Fig. 6h). We next performed a ChIP assay of the potential pSTAT3 binding site in the *Crem* gene promoter and found that mechanical loading induced specific binding of pSTAT3 to the promoter (Fig. 6i, j). In addition, to examine whether mechanical loading promotes pSTAT3 expression via the PGE2-EP4-mediated skeletal interoception pathway, we analyzed the levels of pSTAT3 expression in the PVN of the *EP4*_*Avil*_^–/–^ and *EP4*^*wt*^ mice. By immunostaining of hypothalamic sections we found that expression of pSTAT3 was significantly higher in the PVN of loaded *EP4*^*wt*^ mice compared to the non-loading *EP4*^*WT*^ mice and the stimulation was significantly attenuated in *EP4*_*Avil*_^–/–^ mice (Fig. 6h). Therefore, our data show that mechanical loading activates a PGE2-EP4-mediated skeleton interoceptive pathway to induce activation of PVN pSTAT3 signaling to stimulate the synthesis of CREM.

### Aberrant alterations in PGE2 level leads to ankle osteoarthritis and pain

**Fig. 7:**
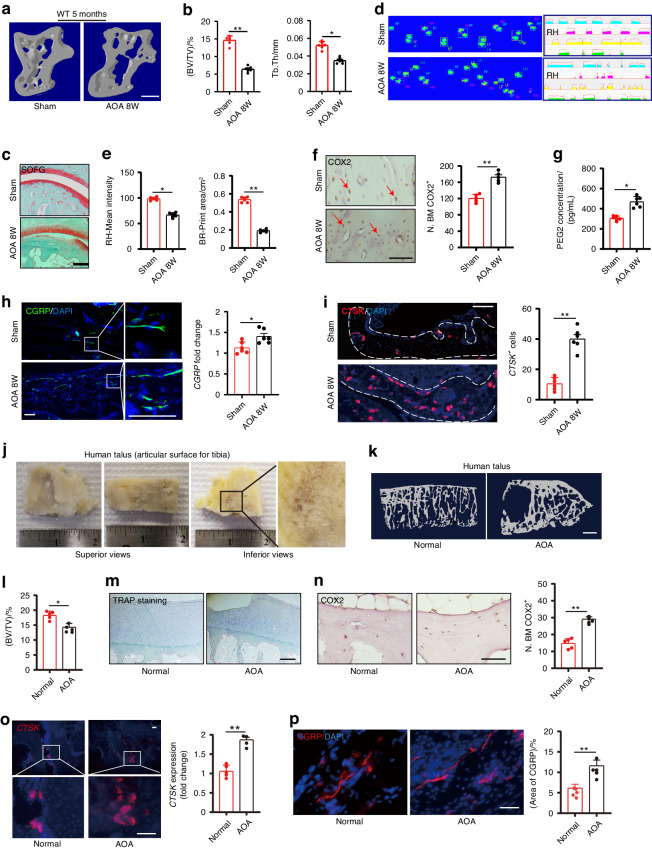
Alteration in PGE2 level leads to Ankle Osteoarthritis and pain through sensory nerve.Representative μCT images **a** and quantitative analysis of trabecular bone fraction (BV/TV) and trabecular bone thickness (Tb. Th) of talus **b** from 20-week-old *C57BL/6* mice with sham or AOA surgery for 8 weeks. Scale bars, 50 μm. **c** Representative images of Safranin Orange and fast green staining in the subchondral bone of talus from 20-week-old *C57BL/6* mice with sham or AOA surgery for 8 weeks (AOA 8W). Scale bars, 50 μm. Representative images of catwalk **d** and analysis **e** of ipsilateral intensity and contact area of right hind paw of 20-week-old *C57BL/6* mice with sham or AOA 8W. **f** Representative images of immunostaining and quantitative analysis of the COX2^+^ (brown) cells in the subchondral bone of talus of 20-week-old *C57BL/6* mice with sham or AOA 8W. Scale bars, 50 μm. **g** ELISA analysis of PGE2 level in the talus of 20-week-old *C57BL/6* mice with sham or AOA 8W. **h** Representative images of immunofluorescence staining and quantitative analysis of the CGRP^+^ sensory nerves (green) in the subchondral bone of talus of 20-week-old *C57BL/6* mice with sham or AOA 8W. Scale bars, 50 μm. **i** Representative images of immunostaining of CTSK and quantitative analysis of CTSK^+^ cells (red) in the subchondral bone of talus of 20-week-old *C57BL/6* mice with sham or AOA 8W. Scale bars, 50 μm. **j** Representative images of human talus samples from end stage of AOA patients with total ankle arthroplasty (TAA). Representative μCT images **k** and quantitative analysis **l** of trabecular bone fraction (BV/TV) of healthy talus and end-stage AOA patient talus. Scale bars, 500 μm. **m** Representative images of immunostaining of TRAP in the subchondral bone of healthy talus and end-stage AOA patient talus. Scale bars, 50 μm. **n** Representative images of immunostaining and quantitative analysis of the COX2^+^ cells (brown) in the subchondral bone of healthy talus and end-stage AOA patient talus. Scale bars, 50 μm. **o** Representative immunofluorescence staining and quantitative analysis of CTSK (red) in the subchondral bone of healthy talus and end-stage AOA patient talus. Scale bars, 50 μm. **p** Representative images of immunofluorescence staining and quantitative analysis of the CGRP^+^ sensory nerves (red) in the subchondral bone of healthy talus and end-stage AOA patient talus. Scale bars, 50 μm. *n* ≥ 5 per group. *P < 0.05, **P < 0.01, and N.S. indicates not significant. Statistical significance was determined by Student’s *t*-test

We next tested whether sensory innervation is initiated by PGE2 and is associated with AOA-associated pain. In mice, we excised the calcaneofibular ligament, the anterior talofibular ligament and the lateral ankle capsule (lateral model), an established mouse model of AOA and ankle pain. Both bone marrow (BV/TV) and trabecular bone parameters were significantly lower in 12-week AOA mice (Fig. 7a, b). By safranin O and fast green (SOFG) staining of the talus we found that a green-stained bone matrix surrounded the cavities in the talus of AOA mice (Fig. 7c), suggesting marked degeneration of the subtalar joint 8 weeks after surgery. Furthermore, ink blot analysis revealed a significant disparity between the percentage of right hind paw ipsilateral intensity and contact area (Fig. 7d, e) of the 2 limbs at 2 months after AOA surgery in wild-type mice relative to sham-surgery controls. The purple waveform represents the mean grip intensity when the right hind paw is walking. COX2 expression in osteocytes was greater in the AOA than in the sham-surgery controls (Fig. 7f). Importantly, the density of CGRP^+^ neurofilaments was markedly higher in AOA mice than in the sham-surgery group (Fig. 7h), and PGE2 levels in the talus of the AOA mice was higher than in the sham controls (Fig. 7g), suggesting that surgery leads to an increase in mechanical loading that in turn leads to greater PGE2 levels. In addition, CTSK expression in osteocytes was significantly greater in the AOA mice compared with sham-surgery mice (Fig. 7i), suggesting that CTSK activity is associated with CGRP^+^ sensory innervation in the talus.

To further test our hypothesis in human disease, we collected 5 surgery specimens from patients with end-stage AOA who underwent total ankle arthroplasty (TAA). These specimens were systemically scanned and analyzed by μCT. The images demonstrated the specimens included the serial sagittal sections (Fig. 7j) and showed that during the end-stage AOA period there was significantly less bone volume compared with the healthy talus (Fig. 7k, l). There was no difference in TRAP^+^ osteoclast expression between the healthy controls and the AOA samples in the talus (Fig. 7m). Importantly, COX2 expression in osteocytes was significantly greater in the AOA samples (*n* = 5) compared to the healthy talus (*n* = 5) (Fig. 7n). Osteocyte expression of CTSK at steady state have been reported in healthy humans^45^, and here by immunostaining for CTSK in the talus from the normal and AOA groups we observed significantly greater CTSK expression in the subchondral bone of talus 8-week after AOA samples compared to the normal healthy control (Fig. 7o). Furthermore, by immunostaining of talus sections we found that there were more CGRP^+^ sensory nerve fibers in the AOA human samples compared to the normal healthy talus (Fig. 7p). Together, these results suggest that elevated PGE2 is associated with AOA-related pain via sensory nerve induction.

## Discussion

The brain was evolved to perceive physical and chemical stimuli in coordination with the activity of multicellular organisms to survive. Gravity is a physical stimulus on Earth. Skeletal interoception could be developed at an early stage during terrestrial animal evolution. In the current study, we demonstrate that skeletal interoception transforms mechanical signaling of weight-bearing bones into biochemical signals in bone cells, and particularly, PGE2 levels in bones are proportional to their weight bearing. When weight bearing changes, for example, via hindlimb unloading in mice, the PGE2 concentrations in various bones change according to changes in their weight bearing. Moreover, deletion of *COX2*, and thus elimination of PGE2 expression, in osteoblast lineage cells or knockout of *Ep4* in sensory nerve blunts bone formation in response to mechanical loading. And sensory deletion of *TrkA* in sensory nerve also significantly reduces mechanical loading-induced bone formation. Moreover, mechanical loading induces CREB phosphorylation in the ARC to inhibit sympathetic *Th* expression in the PVN, thus promoting osteogenesis. Therefore, the hypothalamus perceives elevation of bone PGE2 in response to mechanical loading to maintain bone remodeling, structure, and homeostasis.

Bone formation during remodeling under mechanical loading is an energy-intensive process consuming glucose, ATP, oxygen and free fatty acids^46–48^. Therefore, it is important to provide the resources from different organs for mechanical-induced bone remodeling. We found local mechanical loading induced a significant increase in pCREB expression in the ARC, which was blocked by conditional knockout of osteoblast *COX2* or sensory *EP4*. This suggests that mechanical loading acts along a PGE2-EP4 sensory axis to transmit the change in peripheral status to the hypothalamus to promote a compensatory response. In this manner, proper skeletal interoception is achieved.

We previously showed that elevated PGE2 concentrations in the bone marrow activated the expression of pCREB and the hypothalamic transcriptional co-repressor SMILE. SMILE and pCREB form a heterodimer to bind to the *NPY* promoter to downregulate the expression of hypothalamic NPY and in turn, induce adipose tissue lipolysis for osteoblastic bone formation^11^. Thus, the activation of pCREB after mechanical loading suggests that this stimulus promotes the downregulation of hypothalamus NPY signaling to coordinate the fuel needed for proper bone synthesis. In this way, AgRP^+^ neuron-expressed NPY in the ARC plays a critical role in the central control of bone homeostasis.

PVN is an important component of the hypothalamic-pituitary axis^49^. The neural circuit from the subfornical organ to PVN has been shown to inhibit serum PTH levels and bone formation^50^. Oxytocin and arginine vasopressin peptides produced by the PVN neurons are delivered to the pituitary to control bone formation^51,52^. The heterogeneous neurons of PVN contribute to the differential regulation of bone metabolism. In addition to the neuroendocrine regulation, PVN also involves the autonomic nervous system for the skeletal system^53^. TH^+^ neurons are highly prevalent in the PVN and are key regulators of sympathetic activity^54^. We previously reported that downregulation of TH upon elevated PGE2 levels reduced sympathetic activity outflow to allow for the greater osteoblastic differentiation of mesenchymal stem/stromal cells (MSCs) and increased bone formation^10^. Here, we found that mechanical loading suppresses TH expression in the PVN. Applying the retrograde neural tracer CTB, we found that ARC neurons project to the PVN and that specific chemogenetic inhibition of AgRP^+^ neurons in the ARC significantly blocks mechanical loading-induced TH suppression in the PVN. These data suggest a mechanical loading-ARC (AgRP)-PVN (TH) circuitry acts upstream to regulate TH expression to mediate the mechanical loading interoceptive pathway.

We further identified the mechanistic pathway in the PVN that regulates *Th* expression by ChIP assay. Notably, we found that mechanical loading promotes the elevation of pSTAT3 levels in the PVN, perhaps via the ARC neural circuitry, and that the elevated pSTAT3 in turn binds to the promoter region of *Crem*, the gene that encodes the cAMP response element modulator, CREM, elevating its expression. CREM, in turn, binds to the promoter of *Th* to suppress its expression, thus suppressing sympathetic tone to promote bone formation. Together, these findings show that hypothalamic regulation of NPY and TH activity in response to mechanical loading coordinates energy metabolism with bone structure remodeling. Thus, the brain is essential for the control of mechanical loading-induced bone formation.

Compared to bones in other body locations, we found that under homeostatic conditions in adult mice, the talus contains the highest PGE2 concentration, bone volume fraction, and cortical thickness. HU for 7 days dramatically reduced PGE2 levels, as well as bone mass and cortical bone, in the talus, indicating an active remodeling process in this bone in response to changes in mechanical loading. Interestingly, there are barely detectable TRAP^+^ osteoclasts in the talus from either healthy or AOA mice talus or in talus samples from humans with AOA. Bone remodeling is initiated by osteoclastic bone resorption, which is coupled with osteoblastic bone formation^55–57^. This raises a question of how bone resorption is processed in talus remodeling without osteoclasts. By immunostaining, we found a much higher number of CTSK^+^ osteocytes in mouse talus than in the calcaneus and tibia. The number of CTSK^+^ osteocytes in talus were significantly increased after 7 days of HU or 8 weeks after AOA surgery, and this increase is accompanied by bone loss. Similarly in the human talus, we detected no TRAP^+^ osteoclasts from either patients with AOA or from the healthy controls. However, CTSK was expressed in both healthy and AOA talus, and its levels were significantly higher in the AOA talus and accompanied by significant bone loss compared to the healthy controls. This suggests that in the talus CTSK^+^ osteocytes likely perform the resorptive function usually mediated by osteoclasts. Indeed, the SNS promotes osteocyte-driven bone loss by secretion of extracellular vesicles containing bone-degrading enzymes for perilacunar bone resorption during lactation^58^. It appears that skeletal interoception promotes osteocyte-mediated bone resorption primarily in weight-bearing bones, including cortical bone, whereas osteoclast-mediated bone remodeling occurs in the trabecular bone or in bones that bear less weight. AOA presents a condition that causes pain and disability, and currently, there is no disease-modifying treatment other than orthopedic surgery^59^. The finding of high levels of PGE2 in the talus to maintain its homeostasis through skeletal interoception may explain the painful nature of AOA and shed light on a potential therapy.

**Fig. 8:**
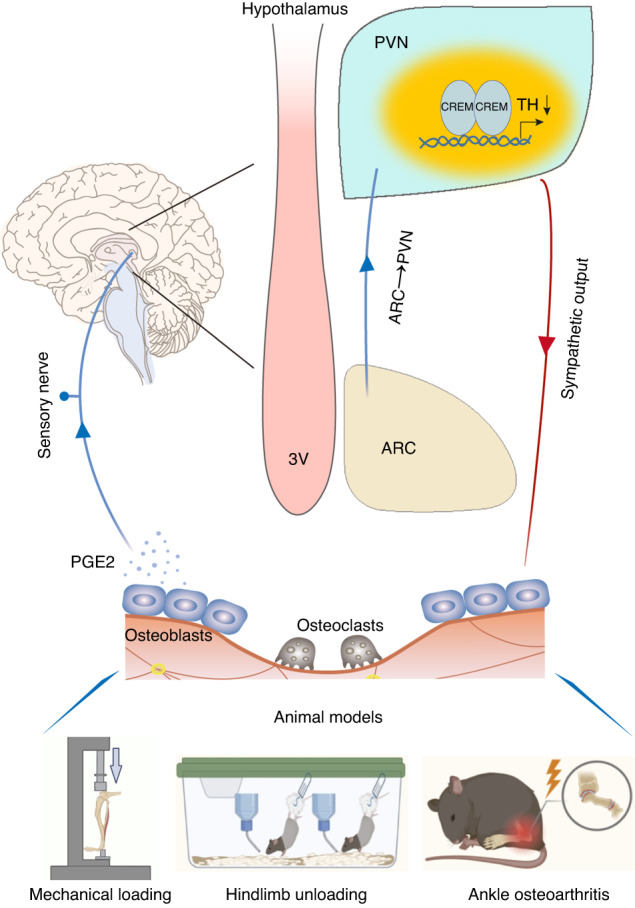
Schematic diagram of PGE2 skeletal interoception regulating bone remodeling in response to mechanical loading.Mechanical loading induces secretion of PGE2, which binds to and activate the EP4 receptor on sensory nerves. The activated sensory nerves transmit the signals to ARC and then to PVN in the hypothalamus where TH transcriptional activity is downregulated. Mechanically, expression of CREM in the PVN is upregulated with mechanical loading, and CREM binds to the TH promoter to its transcription for the changes of sympathetic tone activity. Aberrant mechanical loading in ankle osteoarthritis could lead to pathological changes in talus. Three animal models used in the study: Tibia axial mechanical loading, hindlimb unloading, and ankle osteoarthritis (bottom)

In summary, our results demonstrate that the hypothalamus regulates bone remodeling and structure by perceiving elevated bone PGE2 concentrations that occur in response to mechanical loading. The mechanical loading promotes PGE2/EP4-ARC-PVN skeletal interoceptive ascending pathway from bone to the brain to downregulate sympathetic tone signals for bone remodeling (Fig. 8). It is PGE2 ascending interoceptive signaling modulates hypothalamic tyrosine hydroxylase activity for weight-bearing bone homeostasis. If aberrant loading, particularly in AOA, then this could cause chronic pain, with implications for potential treatment.

## Materials and methods

### Mice and in vivo treatment

The *Advillin-Cre* (*Avil-Cre*) mouse strain was kindly provided by Xingzhong Dong (The Johns Hopkins University, Baltimore, MD). The *Osteocalcin-Cre* (*Ocn-Cre*) mice were obtained from Thomas J. Clemens (The Johns Hopkins University). The *TrkA*^*fl/fl*^ mice were obtained from David D. Ginty (Harvard Medical School, Boston, MA). The *EP4*^*fl/fl*^ mice were obtained from Brian L. Kelsall (National Institutes of Health, Bethesda, MD). The *COX2*^*fl/fl*^ mice were provided by Harvey Herschman (University of California, Los Angeles). The *AgRP-IRES-Cre* mice were purchased from the Jackson Laboratory (012899, Bar Harbor, ME). Heterozygous male *Avil-Cre* mice (female *Avil-Cre* mice were not used for breeding because of the risk of leakage of TrkA protein into the eggs) were crossed with a *TrkA*^*fl/fl*^, *EP4*^*fl/fl*^ mouse. The offspring were intercrossed to generate the following genotypes: *Avil-Cre* (Cre recombinase expressed driven by Advillin promoter), *Avil-Cre::EP4*^*fl/fl*^ (conditional deletion of the *EP4* receptor in Advillin lineage cells, referred to as *EP4*_*Avil*_^*–/–*^ in the text), *Avil-Cre::TrkAfl/fl* (referred to as *TrkA*_*Avil*_^*–/–*^ in the text). Heterozygous *Ocn-Cre* mice were crossed with *COX2*^*fl/fl*^ mouse. The offspring were intercrossed to generate the following genotypes: *Ocn-Cre*, *COX2*^*fl/fl*^, *Ocn-Cre::COX2*^*fl/fl*^ (referred to as *COX2*_*Ocn*_^*−/−*^ in the text) mice. The genotypes of the mice were determined by PCR analyses of genomic DNA, which was extracted from mouse tails within the following primers: *Avil-Cre*: forward: CCCTGTTCACTGTGAGTAGG, reverse: GCGATCCCTGAACATGTCCATC, WT: AGTATCTGGTAGGTGCTTCCAG; *Ocn-Cre*: forward: CAAATAGCCCTGGCAGATTC, reverse: TGATACAAGGGACATCTTCC; *EP4* loxP allele forward: TCTGTGAAGCGAGTCCTTAGGCT, reverse: CGCACTCTCTCTCTCCCAAGGAA； *TrkA* loxP allele forward: AACAGTTTTGAGCATTTTCTATTGTTT, reverse: CAAAGAAAACAGAAGAAAAATAATAC; *COX2* loxP allele forward: AATTACTGCTGAAGCCCACC, reverse: GAATCTCCTAGAACTGACTGG. All mice were maintained at the animal facility of The Johns Hopkins University School of Medicine (Baltimore, MD). We obtained whole blood samples by cardiac puncture immediately after euthanasia. Serum was collected by centrifuge at 1 500 r/min for 15 min and stored at −80°C before analyses. Mice bones, brains were also collected.

The drugs and compounds used in this study are as follows: Clozapine *N*-oxide (CNO, 4936, Tocris, Minneapolis, MN). Dosages and time courses are noted in the corresponding text and figure legends.

### Hindlimb unloading model

When performing hindlimb unloading of mice, place the animals in a bright and clean room. In order to protect the hair on the tail of the mouse, we first gently wrap the tail of the mouse with a sterile dry cotton ball, and then use a soft tape to warp the tail. The tape and the cage were fixed so that the mouse’s front paws could touch the bottom of the cage so that it could eat and drink freely, while the back paws were completely off the ground. This state lasts for 4–5 h every day, and the entire process takes a total of 7 days. It is worth mentioning that we put two mice in a mouse cage at the same time for the experiment, in order to prevent them from developing depression due to long-term hindlimb unloading experiments.

### Mechanical loading

Mechanical loading of 12‐week‐old male mice was performed as previously described^60^. Briefly, the mice were anesthetized with isoflurane (Forane, Baxter International Inc., Deerfield, IL) for the duration of the experiment. The left tibia was axially compressed by fixing the knee and ankle into molded cups on the electromagnetic mechanical actuator (ElectroForce 5500, TA Instruments, New Castle, DE). Loading was applied with a continuous 2‐Hz sinusoidal waveform ranging from 2‐N compressive loading and a 12‐N peak loading for 100 cycles per day on five consecutive days per week. The right tibia served as the internal control. Unrestricted cage activity was allowed between loading bouts. For the time‐course experiments, mice were euthanized after one week or one month of loading (*n* = 5–9 per group). μCT analyses were performed on the mice in the one month loading group.

### Ankle osteoarthritis model

The AOA (Ankle Osteoarthritis) mouse model was established as previously described^61,62^. Briefly, 4-month-old *C57BL/6* female mice (Jackson Laboratory) were anesthetized and underwent AOA or a sham operation of right paw. Postoperative care consisted of an injection of 5.0 mg/kg carprofen (Rimadyl; Zoetis Inc, Parsippany-Troy Hills, NJ) diluted with saline, time under a warming lamp, and visual monitoring at least once every 24 h for 72 h. A 12.5-mg carprofen tablet was administered for pain management, no additional medication was needed. All animals were maintained at the animal facility of the Johns Hopkins University School of Medicine. We obtained whole blood samples by cardiac puncture immediately after euthanasia. Serum was collected by centrifuge at 1 500 r/min for 15 min and stored at −80°C before analyses. Talus of the mice were also collected.

### CatWalk analysis

Gait parameters of freely moving mice were measured using the CatWalk gait analysis system (Noldus Information Technology) as described previously^34^. Briefly, the CatWalk instrument consists of an enclosed walkway with a glass plate floor, a fluorescent lamp that emits light inside the glass plate, a highspeed color video camera, and recording and analysis software to assess the gait of rodents. Each mouse was placed individually in the CatWalk walkway and allowed to walk freely and traverse from one side to the other of the walkway. Mice were trained 3 times before the test. The recordings were made when the room was completely dark, except for the light from the computer screen. Where the mouse paws made contact with the glass plate, light was reflected down and the illuminated contact areas recorded with a high-speed color video camera that was positioned under the glass plate and connected to a computer running the CatWalk software, v7.1. The software automatically labeled all areas containing pixels above the set threshold (7 pixels). These areas were identified and assigned to the respective paws. The recording generated a wide range of parameters, the following 7 of which were analyzed: paw pressure, paw print area, stance phase, swing phase, duty cycle, stride length, and swing speed.

### Stereotaxic injections and neural tracer labeling

Male mice at least 6 weeks of age were anaesthetized with isoflourane, then placed into the stereotaxic apparatus (Stoelting Instruments). The skull was exposed via a small incision and small holes were drilled on skull for viral or tracer injections. A pulled glass pipette with 20–40 μm tip diameter was inserted into the brain for virus or dye delivery. Briefly, 200 nL unilateral injections of pAAV-hSyn-DIO-hM4D(Gi)-mCherry (44362, Addgene) were made in the ARC of *AgRP-Ires-Cre* mice (coordinates, bregma: anterior-posterior, −1.50 mm; dorsal-ventral, −6.00 mm; lateral, ±0.20 mm). For anterograde tracing, 500 nL unilateral injections of AAV9-hSyn-GFP (SL116013, SignaGen) were made in the ARC of *C57BL/6* mice (coordinates, bregma: anterior-posterior, −1.50 mm; dorsal-ventral, −6.00 mm; lateral, ±0.20 mm). For retrograde labeling, 200 nL unilateral injections of cholera toxin subunit B (CTB-Af488, C34775, Invitrogen) were made in the PVN of *C57BL/6* mice (coordinates, bregma: anterior-posterior, −0.80 mm; dorsal-ventral, −4.90 mm; lateral, ±0.25 mm). Postoperative analgesia was provided (ketoprofen, 5 mg/kg). Mice were allowed 1 week to recover and then acclimated to handling for 1 week before the start of any in vivo studies.

### μCT analyses

μCT analyses were performed on the mice in the one month loading group. Mouse bones were harvested, and the soft tissue around the bone was removed, followed by fixation overnight using 4% paraformaldehyde. μCT analyses were performed using a high-resolution μCT scanner (1174, SkyScan, Bruker, Kontich, Belgium). The voltage of the scanning procedure was 65 keV with a 153-μA current. The resolution was set to 9 μm/pixel. Images were reconstructed using NRecon, version 1.6, software (SkyScan) and analyzed using CTAn, version 1.9, software (SkyScan). We used 3-dimensional model visualization software, CTVol, version 2.0 (SkyScan), to analyze the diaphyseal cortical bone and the metaphyseal trabecular bone parameters of the bone. We created cross-sectional images of the femur to perform 2-dimensional analyses of cortical bone and 3-dimensional analyses of trabecular bone. The regions of interest were defined to cover the whole sagittal plane for talus and 0.45 mm in length centered 3 mm proximal to the distal distal tibiofibular junction (TFJ) for tibia, and 0.5 mm in length extended from 0.25 mm distal to the growth plate level in the direction of the metaphysis. The trabecular bone volume fraction (BV/TV), trabecular thickness (Tb.Th), trabecular number (Tb.N) were collected from the 3-dimensional analysis data and used to represent the trabecular bone parameters. 2D structural analyses of cortical bone area (Ct.Ar) and cortical thickness (Ct.Th) were collected to represent cortical bone parameters.

### Histochemistry, immunohistochemistry and immunofluorescence assay

The samples were sectioned at 4 μm or 40 μm intervals using a Microm cryostat (for frozen blocks) or a Paraffin Microtome (for paraffin blocks). We processed 4-μm-thick sections of bone for H&E staining and safranin o (Sigma-Aldrich, S2255) and fast green (Sigma-Aldrich, F7252) staining. TRAP staining was processed following the manufacturer’s protocol (Sigma-Aldrich, 387A-1KT), followed by counterstaining with Methyl Green (Sigma-Aldrich, M884). Briefly, the bone samples were fixed for 4 h with 4% paraformaldehyde at 4 °C and then decalcified at 4 °C using 0.5 mol/L EDTA (pH, 7.4) for 24 h with constant shaking. The samples were dehydrated in 20% sucrose and 2% polyvinylpyrrolidone solution for 24 h and embedded in 8% gelatin (G1890, Sigma-Aldrich) in the presence of 20% sucrose and 2% polyvinylpyrrolidone. Forty μm–thick coronal-oriented sections of bone samples were obtained. For brain section preparation, the whole brain was collected from euthanized mice and fixed with 4% paraformaldehyde for 30 min. Then, the tissue was dehydrated with 20% sucrose for 24 h, followed by 30% sucrose for 24 h and sectioned.

Immunostaining was performed using standard protocol. Briefly, the sections were incubated with primary antibodies to mouse osteocalcin (1:200, M173, Takara Bio), COX2 (1:100, ab15191, Abcam), CTSK (1:200, PA5-102483, Thermo), CGRP (1:100, ab81887, Abcam), pCREB (1:100, ab32096, Abcam), TH (1:200, AB152, Sigma) overnight at 4 °C. A horseradish peroxidase–streptavidin detection kit (Dako, Agilent, Santa Clara, CA) was used in immunohistochemical procedures to detect immuno-activity, followed by counterstaining with hematoxylin (S3309, Dako). Fluorescence-conjugated secondary antibodies were used in immunofluorescent procedures to detect fluorescent signals after counterstaining with DAPI (H-1200, Vector, Burlingame, CA). We used a LSM 780 confocal microscope (Zeiss, Oberkochen, Germany) or an Olympus BX51 microscope (Olympus, Tokyo, Japan) for sample image capturing. Quantitative histomorphometric analysis was performed by using OsteoMeasure XP software (OsteoMetric, Decatur, GA) in a blinded fashion.

### ChIP

ChIP was performed according to instructions from the Pierce Agarose ChIP Kit (26156, Thermo Fisher Scientific, Waltham, MA) with ChIP-grade antibody CREM (sc-390426, Santa Cruz Biotechnology), pSTAT3 (9145, Cell Signaling Technology). Briefly, we added cells with formaldehyde to cross-link proteins to DNA, and the cells were lysed in 1.5-mL lysis buffer (50 mmol/L HEPES, pH 7.5, 140 mmol/L NaCl; 1 mmol/L EDTA; 1% Triton X-100; 0.1% sodium deoxy cholate; 0.1% sodium dodecyl sulfate). Cell lysates were sonicated at 2 s on/15 s off for 3 rounds using a Bioruptor ultrasonic cell disruptor (Diagenode, Denville, NJ) to shear genomic DNA to a mean fragment size of 150 to 250 bp. Of the sample, 1% was removed for use as an input control. ChIP was performed according to the protocol provided by the Simple Chip Enzymatic Chromatin IP Kit (Cell Signaling Technology) using antibodies. Anti-RNA polymerase II and control IgG were used as positive and negative controls, respectively. After washing and de-crosslinking, the precipitated DNA was purified using a QIA quick PCR purification kit (Qiagen, Hilden, Germany).

### Quantitative real-time polymerase chain reaction (qPCR)

Total RNA was purified from tissues using TRIzol (15596026, Invitrogen, Carlsbad, CA) following the manufacturer’s protocol. CREM primer: 5’-ATGGCTGTAACTGGAGATGAA-3’ (forward) and 5’-GTGGCAAAGCAGTAGTAGGA-3’ (reverse). We performed qPCR using the Taq SYBR Green Power PCR Master Mix (A25777, Invitrogen) on a CFX Connect instrument (Bio-Rad Laboratories, Hercules, CA); Gapdh amplification was used as an internal control. Dissociation curve analysis was performed for every experiment. Sequences of the primers used for each gene are available on request.

### ELISA and Western Blot

PGE2 concentrations in the bone marrow were determined by PGE2 ELISA kit (Cayman Chemical, 514010) according to the manufacturer’s protocol. We euthanized mice, collected the bones from different parts. The spine was taken from the fourth to fifth lumbar vertebrae. We collected the complete femur and tibia to measure the overall content of PGE2 in both the femur and tibia. Additionally, we separately collected the trabecular, cortical, and cancellous bone from the femur, as well as the subchondral bone from the tibia, to assess the content of PGE2 in different areas. Then we centrifuged the samples for 15 min at 800 × *g* at 4 °C to obtain bone marrow supernatants, which were stored at –80 °C until ELISA.

Western blot analyses were conducted on the protein of lysates from the hypothalamus of mice. The supernatants of lysates were collected after centrifugation and separated by sodium dodecyl sulfate‐polyacrylamide gel electrophoresis (SDS‐PAGE), and then blotted on the nitrocellulose blotting membranes (MilliporeSigma, Burlington, MA). The primary antibody for CREM (1:1 000, sc-390426, Santa Cruz Biotechnology) and β‐Actin (1:3 000, A2228, Sigma) was applied for incubation.

### Statistical analysis

All data analyses were performed using SPSS, version 15.0, software (IBM Corp., Armonk, NY). Data are presented as means ± standard errors of the mean. For comparisons between 2 groups, we used 2-tailed Student *t*-tests. For comparisons among multiple groups, we used 2-way analysis of variance. All relevant data are available from the authors.

### Study approval

All human samples were obtained from patients undergoing total talus replacement surgery in the Department of Orthopedics Surgery at JHU Hospital. The patients’ consent, as well as approval of the local ethics committees, were obtained before harvesting human tissue samples. The experimental protocol was reviewed and approved by the Institutional Animal Care and Use Committee of Johns Hopkins University.

All animal experiments were performed in accordance with NIH policies on the use of laboratory animals. All experimental protocols were approved by the Animal Care and Use Committee of The Johns Hopkins University.

## Data Availability

All data associated with this study are present in the paper.
